# Pragmatic cardiovascular-kidney-metabolic burden categories and 5-year all-cause mortality in Vietnamese outpatients: A retrospective cohort study

**DOI:** 10.1371/journal.pone.0354433

**Published:** 2026-07-22

**Authors:** Duc Khanh Nguyen, Thanh Tuan Tran, Van Sy Hoang

**Affiliations:** 1 School of Medicine, University of Medicine and Pharmacy at Ho Chi Minh City, Ho Chi Minh City, Vietnam; 2 Department of Cardiology, Cho Ray Hospital, Ho Chi Minh City, Vietnam; The Chinese University of Hong Kong, HONG KONG

## Abstract

**Background:**

The American Heart Association introduced cardiovascular-kidney-metabolic (CKM) health as an integrated framework for metabolic, kidney, and cardiovascular risk. However, full AHA CKM staging requires variables that are often unavailable in retrospective outpatient datasets, and outcome data from Southeast Asia remain limited.

**Objective:**

To evaluate whether pragmatic CKM burden categories derived from routinely available baseline diagnoses identify Vietnamese outpatients at higher risk of 5-year all-cause mortality.

**Methods:**

We performed a retrospective cohort analysis of 480 adult outpatients recruited from 01 January 2016–31 December 2016 in Ho Chi Minh City, Vietnam. The de-identified dataset for this secondary analysis was accessed on 31 March 2024. Participants were classified into four mutually exclusive pragmatic CKM burden categories: Category A, no documented metabolic-risk diagnosis, chronic kidney disease (CKD), or coronary artery disease (CAD); Category B, documented metabolic-risk diagnosis only; Category C, CKD or CAD, but not both; and Category D, concomitant CKD and CAD. These categories are AHA-informed but are not official AHA CKM stages. The primary endpoint was 5-year all-cause mortality, with administrative censoring at 5 years. Logistic regression was the primary inferential model; Kaplan-Meier, log-rank, and Cox models were used as complementary time-to-event analyses. To support privacy-preserving data sharing, adjusted models used prespecified age groups (<40, 40–60, and >60 years) and sex rather than exact individual ages.

**Results:**

Most participants were aged 40–60 years (331/480, 69.0%) and 204 (42.5%) participants were men. The category distribution was as follows: Category A, 24 (5.0%), Category B, 252 (52.5%), Category C, 180 (37.5%), Category D, 24 (5.0%). Over 2243.1 person-years of follow-up within the 5-year analysis horizon, 64 deaths occurred (13.3%). Five-year mortality was 0/24 (0.0%) in Category A, 33/252 (13.1%) in Category B, 24/180 (13.3%) in Category C, 7/24 (29.2%) in Category D. Kaplan-Meier curves differed across the four categories (log-rank p = 0.036). Compared with Categories A + B, Category D had higher unadjusted odds of 5-year mortality (OR 3.03, 95% CI 1.17–7.86; p = 0.022) and remained elevated after age-group and sex adjustment (OR 2.87, 95% CI 1.04–7.94; p = 0.042). Category C was not associated with higher adjusted 5-year mortality.

**Conclusions:**

In this Vietnamese outpatient cohort, pragmatic CKM burden categories identified a subgroup with combined CKD and CAD that had the highest absolute 5-year mortality. The age-group- and sex-adjusted estimate remained elevated for this small subgroup, although confidence intervals were wide. These categories should not be interpreted as official AHA CKM stages, and prospective validation with complete CKM phenotyping is needed.

## Introduction

Cardiovascular disease, chronic kidney disease (CKD), and metabolic disorders frequently co-occur and worsen one another through shared risk factors and bidirectional pathophysiology. The American Heart Association (AHA) recently introduced the cardiovascular-kidney-metabolic (CKM) health construct to support integrated prevention, risk prediction, and coordinated care across this interrelated disease continuum [1,2].

The official AHA CKM framework includes stages 0–4 and requires information on adiposity, metabolic risk factors, kidney disease severity, subclinical cardiovascular disease, and clinical cardiovascular disease [1,2]. In many retrospective outpatient cohorts, however, important elements such as albuminuria, waist circumference, coronary artery calcium, and systematic subclinical cardiovascular disease assessment are unavailable. Analyses in such settings therefore require transparent operational definitions that avoid implying full adherence to official AHA staging.

Recent population studies have shown that advanced CKM stages or greater CKM component burden are common and associated with adverse outcomes [3–6]. However, evidence from Southeast Asian outpatient settings remains limited. Vietnamese outpatients may differ from population-based cohorts in referral patterns, baseline cardiovascular disease prevalence, kidney disease ascertainment, and long-term follow-up.

We therefore analyzed a Vietnamese outpatient cohort recruited from 01 January 2016–31 December 2016. In response to concerns about the feasibility of direct 10-year follow-up for all participants, we revised the primary endpoint to 5-year all-cause mortality, a horizon compatible with the recruitment and data access dates. Our aims were to describe pragmatic CKM burden categories based on routinely available baseline variables and to evaluate their association with 5-year all-cause mortality.

## Materials and methods

### Study design and reporting

This study is a retrospective cohort analysis and secondary use of an observational outpatient cohort. Reporting follows the Strengthening the Reporting of Observational Studies in Epidemiology (STROBE) statement [7].

### Study population and setting

The parent cohort was recruited from 01 January 2016–31 December 2016 at outpatient clinics in Ho Chi Minh City, Vietnam. The recruitment start date corresponds to the beginning of the approved study period documented in the ethics approval. Adult outpatients who underwent baseline clinical evaluation and laboratory testing were enrolled during this period. For the present secondary analysis, we included participants with complete baseline variables required to assign pragmatic CKM burden category and with recorded follow-up time and vital status. No participants were excluded after applying these complete-case analytic criteria in the de-identified dataset used for this revision.

The de-identified dataset was accessed for research purposes on 31 March 2024. Exact individual calendar enrollment dates were not available in the secondary analysis dataset. The authors did not have access to information that could identify individual participants during or after data collection for the present analysis. For public data sharing, exact age was replaced with prespecified age categories (<40, 40–60, and >60 years), and specific calendar dates and direct identifiers were not shared.

### Pragmatic CKM burden categorization

We operationalized four mutually exclusive pragmatic CKM burden categories using variables available in the cohort. A documented metabolic-risk diagnosis was defined as baseline history of hypertension, diabetes mellitus, and/or dyslipidemia. CKD was defined as documented CKD history and/or estimated glomerular filtration rate (eGFR) <60 mL/min/1.73 m², consistent with standard CKD threshold definitions [8]. CAD was defined as documented coronary artery disease history.

These categories are AHA-informed because they use metabolic, kidney, and cardiovascular domains emphasized by the CKM construct, but they are not equivalent to the official AHA CKM stages. Specifically, the cohort did not include waist circumference, albuminuria, systematic subclinical cardiovascular disease testing, noncoronary cardiovascular disease phenotyping, or the full AHA stage 4a/4b subcategorization.

**Table:** 

Pragmatic category	Operational definition in this study	Relationship to official AHA CKM framework
Category A	No documented metabolic-risk diagnosis, CKD, or CAD	Most similar to low documented CKM burden; not identical to official stage 0 because adiposity and subclinical disease were not fully assessed
Category B	Documented hypertension, diabetes mellitus, and/or dyslipidemia without CKD or CAD	Reflects metabolic-risk diagnosis burden; not identical to official stages 1–2
Category C	CKD or CAD, but not both, regardless of documented metabolic-risk diagnoses	Single kidney or coronary disease domain; not identical to official stage 3 or 4 because subclinical and noncoronary CVD were unavailable
Category D	Concomitant CKD and CAD	Highest observed cardio-kidney burden in this dataset; not equivalent to official AHA stage 4a/4b

### Outcome and follow-up

The primary outcome for this revised analysis was 5-year all-cause mortality. Follow-up time was calculated from baseline to death or last recorded contact and was administratively censored at 5 years for the primary analysis. A fixed 5-year horizon was chosen because participants recruited as late as 31 December 2016 still had more than 7 years of potential calendar follow-up by the dataset access date of 31 March 2024. We therefore no longer present the primary outcome as directly observed 10-year mortality.

Deaths recorded at 0.01 years were retained in the primary analysis and excluded in a sensitivity analysis because these events may represent very early post-baseline deaths or rounding of event times. We also performed a sensitivity analysis using the full recorded follow-up time and death indicator, but this analysis was interpreted as recorded follow-up mortality rather than a fixed 10-year endpoint.

### Statistical analysis

Continuous variables are summarized as mean ± SD or median (interquartile range), and categorical variables as counts (percentages). Kaplan-Meier curves were used to visualize 5-year survival across Categories A-D and were compared with the log-rank test. Numbers at risk are presented below the Kaplan-Meier plot.

Because Category A had no 5-year deaths, Categories A and B were combined as the reference group for regression analyses to avoid zero-event instability. The primary inferential model was logistic regression for 5-year all-cause mortality, reported as odds ratios (ORs) with 95% confidence intervals (CIs). Models were fitted unadjusted and adjusted for age group and sex. Cox proportional hazards models over the administratively censored 5-year follow-up were used as complementary time-to-event analyses and are reported as hazard ratios (HRs). Category burden was also evaluated as an ordinal score from A = 0 to D = 3. Exploratory age-stratified summaries were produced for participants aged <40, 40–60, and >60 years; formal age-stratum-specific modeling was not emphasized because several cells had zero events.

All analyses were two-sided with p < 0.05 considered statistically significant. Analyses were conducted in Python using pandas, statsmodels, scipy, and matplotlib. The revised analysis script is provided as supporting analysis code.

### Ethics

The parent study protocol was approved by the Biomedical Research Ethics Committee, University of Medicine and Pharmacy at Ho Chi Minh City (approval No. 454/ĐHYD-HĐ; 30 December 2015). Written informed consent was obtained from all participants in the parent cohort. The present work is a secondary analysis of de-identified data, and no new participant contact or intervention was performed.

## Results

Participant flow is summarized in S1 Fig. The analytic cohort included 480 participants with complete baseline variables for pragmatic CKM categorization and recorded follow-up time and vital status.

Most participants were aged 40–60 years (331/480, 69.0%), and 204 (42.5%) participants were men. The category distribution was as follows: Category A, 24 (5.0%), Category B, 252 (52.5%), Category C, 180 (37.5%), Category D, 24 (5.0%). Within Category C, CAD alone accounted for 96 participants (53.3%) and CKD alone for 84 participants (46.7%). Baseline characteristics by pragmatic CKM burden category are summarized in Table 1. In response to PLOS human-participant data-sharing guidance, exact ages are not shared in the public dataset and Table 1 presents age only in prespecified groups.

**Table 1:** Baseline characteristics by pragmatic CKM burden category.

Characteristic	Overall	Category A	Category B	Category C	Category D
Age < 40 years	50 (10.4%)	3 (12.5%)	28 (11.1%)	19 (10.6%)	0 (0.0%)
Age 40–60 years	331 (69.0%)	15 (62.5%)	182 (72.2%)	122 (67.8%)	12 (50.0%)
Age > 60 years	99 (20.6%)	6 (25.0%)	42 (16.7%)	39 (21.7%)	12 (50.0%)
BMI (kg/m²)	26.3 ± 3.3	25.9 ± 1.3	26.3 ± 3.2	26.4 ± 3.8	26.5 ± 2.0
eGFR (mL/min/1.73 m²)	74.0 ± 13.7	91.8 ± 8.0	77.3 ± 10.4	69.4 ± 15.0	57.0 ± 1.9
LVEF (%)	55.7 ± 9.1	56.6 ± 5.9	55.6 ± 9.6	55.8 ± 8.7	55.3 ± 10.6
Men	204 (42.5%)	0 (0.0%)	96 (38.1%)	90 (50.0%)	18 (75.0%)
Hypertension	306 (63.7%)	0 (0.0%)	156 (61.9%)	132 (73.3%)	18 (75.0%)
Diabetes mellitus	114 (23.8%)	0 (0.0%)	78 (31.0%)	36 (20.0%)	0 (0.0%)
Dyslipidemia	306 (63.7%)	0 (0.0%)	174 (69.0%)	108 (60.0%)	24 (100.0%)
Current smoking	162 (33.8%)	6 (25.0%)	114 (45.2%)	36 (20.0%)	6 (25.0%)
History of CAD	120 (25.0%)	0 (0.0%)	0 (0.0%)	96 (53.3%)	24 (100.0%)
History of CKD/eGFR < 60	108 (22.5%)	0 (0.0%)	0 (0.0%)	84 (46.7%)	24 (100.0%)
hs-CRP (mg/L)	3.15 (2.10-5.30)	2.20 (1.53-2.55)	3.70 (2.10-5.72)	3.15 (2.18-5.72)	3.35 (1.98-4.50)

Values are mean ± SD, median (interquartile range), or n (%), as appropriate. Age is shown only in prespecified categories to protect participant privacy. CAD, coronary artery disease; CKD, chronic kidney disease; CKM, cardiovascular-kidney-metabolic; eGFR, estimated glomerular filtration rate; hs-CRP, high-sensitivity C-reactive protein; LVEF, left ventricular ejection fraction.

**Fig 1:**
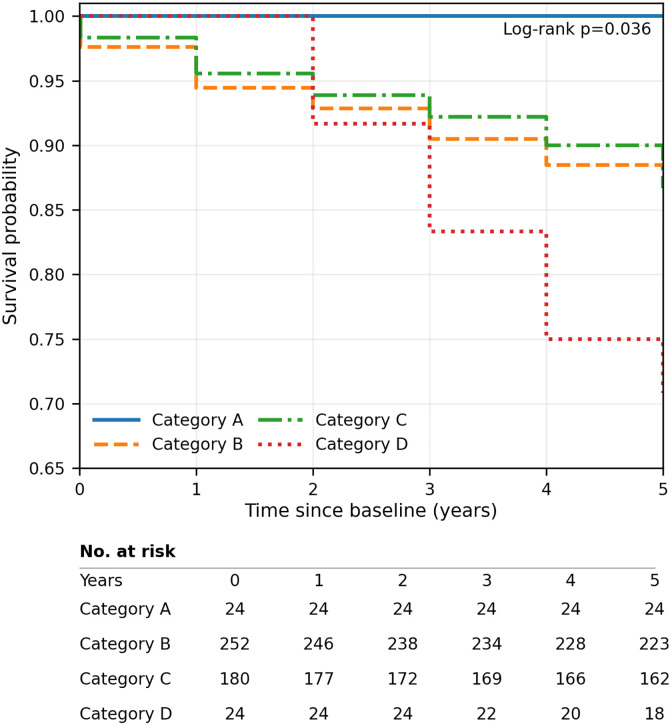
Kaplan-Meier survival curves for 5-year all-cause mortality by pragmatic CKM burden category. The x-axis shows time since baseline in years and the y-axis shows survival probability. Numbers at risk are shown below the plot. Log-rank p = 0.036. CKM, cardiovascular-kidney-metabolic.

Over 2243.1 person-years of follow-up within the 5-year analysis horizon, 64 deaths occurred (13.3%). Five-year mortality increased most clearly in the combined CKD and CAD category: Category A, 0/24 (0.0%); Category B, 33/252 (13.1%); Category C, 24/180 (13.3%); Category D, 7/24 (29.2%). Kaplan-Meier curves over the 5-year horizon differed across Categories A-D (log-rank p = 0.036) (Fig 1).

Because Category A had no 5-year deaths, Categories A and B were combined as the reference group for regression analyses. In unadjusted logistic regression, Category D was associated with higher 5-year mortality compared with Categories A + B, whereas Category C was not. After adjustment for age group and sex, the estimate for Category D remained elevated, although the confidence interval was wide. Cox time-to-event models over the 5-year horizon showed a similar pattern (Table 2).

**Table 2:** Association between pragmatic CKM burden category and 5-year all-cause mortality.

Comparison	5-year mortality	Unadjusted OR (95% CI); p	Age-group- and sex-adjusted OR (95% CI); p	Age-group- and sex-adjusted Cox HR (95% CI); p
Category C vs Categories A + B	24/180 (13.3%)	1.13 (0.65–1.99); p = 0.664	1.17 (0.66–2.09); p = 0.585	1.14 (0.67–1.95); p = 0.619
Category D vs Categories A + B	7/24 (29.2%)	3.03 (1.17–7.86); p = 0.022	2.87 (1.04–7.94); p = 0.042	2.51 (1.05–6.02); p = 0.039
Per category increase (A = 0 to D = 3)			1.56 (1.04–2.34); p = 0.031	1.49 (1.03–2.15); p = 0.035

CI, confidence interval; HR, hazard ratio; OR, odds ratio. Categories A and B were combined as the reference category for regression because Category A had zero 5-year deaths. Adjusted models used age group (<40, 40–60, > 60 years) and sex.

In a sensitivity analysis excluding nine deaths recorded at 0.01 years, the age-group- and sex-adjusted association for Category D became stronger (OR 3.84, 95% CI 1.34–11.01; p = 0.012). In a sensitivity analysis using full recorded follow-up rather than a fixed 5-year horizon, Category D was associated with higher mortality after age-group and sex adjustment (Cox HR 2.44, 95% CI 1.25–4.77; p = 0.009).

Exploratory age-stratified summaries showed no 5-year deaths among participants aged <40 years. Among participants aged 40–60 years, 5-year mortality was similar in Categories A + B and C and no deaths occurred in the small Category D subgroup. Among participants aged >60 years, mortality was highest in Category D (7/12, 58.3%). These exploratory findings are reported in the supporting analysis tables and should be interpreted cautiously because of sparse cells.

## Discussion

In this revised analysis of Vietnamese outpatients, we replaced the original fixed 10-year mortality claim with a 5-year all-cause mortality endpoint and reanalyzed the data accordingly. The combined CKD and CAD group had the highest absolute 5-year mortality, while Categories B and C had similar 5-year event proportions. After age-group and sex adjustment, the Category D estimate remained elevated, but the confidence interval was wide because this subgroup and the number of 5-year deaths were small.

These findings are broadly consistent with the CKM concept that coexisting metabolic, kidney, and cardiovascular disease domains identify higher-risk patients. Large population studies have reported progressively higher mortality across advancing official CKM stages [5,6]. Our study differs because it used pragmatic burden categories rather than official AHA staging, but the high absolute risk observed in participants with concomitant CKD and CAD supports the clinical importance of recognizing combined cardio-kidney disease burden in outpatient care.

The attenuation and imprecision of associations after adjustment are clinically plausible. Age group was a strong predictor of 5-year mortality, and participants in the highest-burden category were older and more often male. The exploratory age-stratified analysis suggested that the mortality gradient was most apparent among participants older than 60 years, but the small number of events and zero-event cells preclude definitive subgroup inference.

This revision also clarifies the relation between our categories and the official AHA CKM framework. We no longer use numerical CKM stage labels because the official AHA framework includes stages 0–4 and stage 4 subcategories, whereas our dataset did not contain all required variables. In particular, lack of albuminuria, waist circumference, systematic subclinical CAD testing, coronary calcium imaging, and noncoronary cardiovascular disease phenotyping could have caused under-ascertainment of higher CKM burden. Some participants classified as Category B may have had unmeasured subclinical cardiovascular disease or kidney damage, which could attenuate observed differences between Categories B and C.

This study has additional limitations. First, it was a retrospective secondary analysis from an outpatient cohort in a single urban region, limiting causal inference and generalizability. Second, metabolic risk was based on documented diagnoses of hypertension, diabetes mellitus, or dyslipidemia; undiagnosed or inconsistently recorded conditions may have led to misclassification. Third, exact individual calendar enrollment dates were not available in the de-identified dataset, which is why we selected a 5-year fixed horizon rather than retaining the original 10-year endpoint. Fourth, exact age is not shared in the public dataset to protect participant privacy; therefore, the reproducible public analysis uses age-group adjustment rather than exact-age adjustment. Fifth, medication use, treatment intensity, socioeconomic factors, albuminuria, cause-specific mortality, and longitudinal changes in CKM status were unavailable. Finally, Category D included only 24 participants, leading to wide confidence intervals.

Despite these limitations, the study provides transparent exploratory evidence from a Southeast Asian outpatient setting and demonstrates how a retrospective dataset can be aligned with the CKM concept without implying full AHA staging. Future studies should use prospective recruitment, standardized follow-up, complete CKM phenotyping, and sufficient sample size to evaluate official AHA CKM stages and stage-guided interventions.

## Conclusions

In Vietnamese outpatients, pragmatic CKM burden categories identified a small subgroup with concomitant CKD and CAD that had the highest absolute 5-year all-cause mortality. The age-group- and sex-adjusted estimate remained elevated, and sensitivity analyses supported higher risk in the combined CKD and CAD group, but estimates were imprecise because of sparse events. These categories are AHA-informed but are not official AHA CKM stages; prospective validation with complete CKM phenotyping is warranted.

## Supporting information

S1 Fig STROBE flow diagram of participant selection and primary analytic cohort. (TIF)

S2 Checklist STROBE checklist for the cohort study. (DOCX)

S3 Table Supporting analysis tables for age-stratified and sensitivity analyses. (DOCX)

S4 Code Revised analysis script used to derive the 5-year endpoint and regression results. (PY)
