# Supplementary material for: Pragmatic cardiovascular-kidney-metabolic burden categories and 5-year all-cause mortality in Vietnamese outpatients: A retrospective cohort study
Source: PLoS One. 2026 Jul 22;21(7):e0354433. doi: 10.1371/journal.pone.0354433 (PMC13390809; doi:10.1371/journal.pone.0354433)
Supplement: S2 Checklist — (DOCX) [file pone.0354433.s002.docx]

# STROBE checklist (cohort study) - revised page and line number version

Reference manuscript: 03_Manuscript_CLEAN.docx

| **Section** | **Item** | **Recommendation** | **Reported in revised manuscript** | **Notes/comments** |
| --- | --- | --- | --- | --- |
| Title/Abstract | 1a | Indicate the study design with a commonly used term in the title or abstract | Title and Abstract; p.1, lines 1-38 | Retrospective cohort study stated |
| Title/Abstract | 1b | Provide an informative and balanced summary of what was done and found | Abstract; p.1, lines 11-38 | Revised to 5-year endpoint and cautious conclusion |
| Introduction | 2 | Explain the scientific background and rationale | Introduction; pp.1-2, lines 42-57 | CKM rationale and Southeast Asian evidence gap |
| Introduction | 3 | State specific objectives, including prespecified hypotheses | Introduction; p.2, lines 58-62 | Objectives revised to 5-year all-cause mortality |
| Methods | 4 | Present key elements of study design early in the paper | Study design and reporting; p.2, lines 66-68 | Retrospective cohort and secondary analysis stated |
| Methods | 5 | Describe setting, locations, and relevant dates, including recruitment, exposure, follow-up, and data collection | Study design; Study population; Outcome; pp.2-3, lines 66-76 and 90-94 | Recruitment dates, data access date, and 5-year administrative censoring reported |
| Methods | 6a | Give eligibility criteria, sources and methods of participant selection, and follow-up methods | Study population and setting; p.2, lines 70-76; Outcome; p.3, lines 90-94 | Adult outpatients; complete baseline/follow-up data; vital status and follow-up time |
| Methods | 6b | For matched studies, give matching criteria and number of exposed/unexposed | Not applicable | No matched design |
| Methods | 7 | Clearly define outcomes, exposures, predictors, confounders, and effect modifiers | Pragmatic CKM burden categorization; Outcome; Statistical analysis; pp.2-4, lines 77-111 | Exposure categories A-D; 5-year mortality; age group and sex; age strata |
| Methods | 8 | Give data sources and details of assessment or measurement | Study population and setting; Pragmatic CKM burden categorization; pp.2-3, lines 70-89 | Baseline diagnoses, eGFR, CAD/CKD definitions, vital status |
| Methods | 9 | Describe efforts to address potential sources of bias | Statistical analysis; Discussion limitations; pp.3-6, lines 95-111 and 169-178 | Complete-case analysis, cautious interpretation, misclassification limitations |
| Methods | 10 | Explain how study size was arrived at | Study population and setting; p.2, lines 70-76 | Available complete-case parent cohort; no additional sampling |
| Methods | 11 | Explain how quantitative variables were handled and why groupings were chosen | Pragmatic CKM burden categorization; Statistical analysis; pp.2-4, lines 77-111 | eGFR threshold; category burden score A=0 to D=3; age strata |
| Methods | 12a | Describe all statistical methods including confounding control | Statistical analysis; pp.3-4, lines 95-111 | Logistic regression primary; KM/log-rank/Cox complementary; age-group/sex adjustment |
| Methods | 12b | Describe methods used to examine subgroups and interactions | Statistical analysis; p.4, lines 106-108; Results; p.5, lines 147-151 | Exploratory age-stratified summaries <40, 40-60, >60 |
| Methods | 12c | Explain how missing data were addressed | Study population and setting; p.2, lines 70-76 | Complete baseline/follow-up data required for inclusion; no missing values in analytic dataset |
| Methods | 12d | Explain how loss to follow-up was addressed | Outcome and follow-up; p.3, lines 90-94 | Administrative censoring at 5 years; rounded follow-up times in public dataset; full recorded follow-up sensitivity |
| Methods | 12e | Describe sensitivity analyses | Statistical analysis; pp.3-4, lines 95-111; Results; p.5, lines 143-146 | Excluding deaths at 0.01 years; full recorded follow-up Cox sensitivity |
| Results | 13a | Report numbers of individuals at each stage of study | Results; p.4, lines 118-123; S1 Fig | N=480; category counts |
| Results | 13b | Give reasons for non-participation at each stage | Study population; p.2, lines 70-76; S1 Fig | No exclusions after complete-case analytic eligibility |
| Results | 13c | Consider use of a flow diagram | S1 Fig; Supporting information captions; p.7, lines 228-230 | Flow diagram supplied |
| Results | 14a | Give characteristics of study participants and information on exposures/confounders | Results; p.4, lines 120-127; Table 1 | Baseline characteristics by category |
| Results | 14b | Indicate number of participants with missing data | Study population; p.2, lines 70-76; S1 Fig | Complete analytic dataset; public dataset privacy-hardened with age categories and no direct identifiers |
| Results | 14c | Summarize follow-up time | Results; p.4, line 128; Supporting analysis tables | Five-year person-years reported |
| Results | 15 | Report numbers of outcome events or summary measures over time | Results; pp.4-5, lines 128-134; Fig 1 | 64 deaths by 5 years; category mortality and KM figure |
| Results | 16a | Give unadjusted and adjusted estimates with precision and state confounders | Results; p.5, lines 135-146; Table 2 | Unadjusted and age-group/sex-adjusted ORs/HRs with 95% CI and p values |
| Results | 16b | Report category boundaries when continuous variables were categorized | Pragmatic CKM burden categorization; p.3, lines 79-83 | eGFR <60 threshold; age categories <40, 40-60, >60 listed |
| Results | 16c | Translate relative risk into absolute risk for a meaningful period | Results; pp.4-5, lines 128-130; Table 2 | Five-year absolute mortality by category |
| Results | 17 | Report other analyses, e.g., subgroup and sensitivity analyses | Results; p.5, lines 143-151; Supporting analysis tables | Sensitivity and age-stratified summaries |
| Discussion | 18 | Summarize key results with reference to objectives | Discussion; p.5, lines 153-157 | Main revised findings and precision noted |
| Discussion | 19 | Discuss limitations, potential bias, and imprecision | Discussion; p.6, lines 169-178 | Operational categories, missing subclinical CAD/albuminuria, small category D, residual confounding |
| Discussion | 20 | Provide a cautious overall interpretation | Discussion and Conclusions; pp.5-6, lines 153-190 | Avoids overclaiming official AHA staging or direct 10-year endpoint |
| Discussion | 21 | Discuss generalizability | Discussion; p.6, lines 179-183 | Single urban outpatient cohort; prospective validation needed |
| Other | 22 | Give funding source and role of funders | Submission system / cover letter | Funding and financial disclosure entered in PLOS submission system |

Privacy note: Following PLOS human-participant data guidance, exact individual age has been removed from the public dataset and Table 1 now reports age only in prespecified categories. Reproducible adjusted models use age group and sex.
