# Supplementary material for: Pragmatic cardiovascular-kidney-metabolic burden categories and 5-year all-cause mortality in Vietnamese outpatients: A retrospective cohort study
Source: PLoS One. 2026 Jul 22;21(7):e0354433. doi: 10.1371/journal.pone.0354433 (PMC13390809; doi:10.1371/journal.pone.0354433)
Supplement: S3 Table — (DOCX) [file pone.0354433.s003.docx]

# Supporting analysis tables for revised CKM burden manuscript

S1 Table. Five-year outcomes by pragmatic CKM burden category.

| Category | N | Five-year deaths | Five-year mortality | Five-year person-years / rate per 100 person-years |
| --- | --- | --- | --- | --- |
| Category A | 24 | 0 | 0.0% | 120.0 / 0.00 |
| Category B | 252 | 33 | 13.1% | 1169.1 / 2.82 |
| Category C | 180 | 24 | 13.3% | 846.0 / 2.84 |
| Category D | 24 | 7 | 29.2% | 108.0 / 6.48 |

S2 Table. Exploratory age-stratified five-year mortality.

| Age group | Regression category | N | Five-year deaths | Five-year mortality |
| --- | --- | --- | --- | --- |
| <40 years | A+B | 31 | 0 | 0.0% |
| <40 years | C | 19 | 0 | 0.0% |
| <40 years | D | 0 | 0 | NA |
| 40-60 years | A+B | 197 | 30 | 15.2% |
| 40-60 years | C | 122 | 18 | 14.8% |
| 40-60 years | D | 12 | 0 | 0.0% |
| >60 years | A+B | 48 | 3 | 6.2% |
| >60 years | C | 39 | 6 | 15.4% |
| >60 years | D | 12 | 7 | 58.3% |

Note: Formal stratum-specific modeling was not emphasized because several cells had zero deaths. Chi-square p-values across Categories A+B, C, and D were not estimable for <40 years, p=0.345 for 40-60 years, and p<0.001 for >60 years.

S3 Table. Sensitivity analyses using age-group- and sex-adjusted models.

| Sensitivity analysis | Comparison | Adjusted estimate (95% CI); p |
| --- | --- | --- |
| Excluding deaths recorded at 0.01 years; 5-year age-group- and sex-adjusted logistic model | Category C vs A+B | 1.31 (0.71-2.44); p=0.392 |
| Excluding deaths recorded at 0.01 years; 5-year age-group- and sex-adjusted logistic model | Category D vs A+B | 3.84 (1.34-11.01); p=0.012 |
| Full recorded follow-up; age-group- and sex-adjusted Cox model (sensitivity, not fixed 10-year endpoint) | Category C vs A+B | 1.53 (1.03-2.28); p=0.036 |
| Full recorded follow-up; age-group- and sex-adjusted Cox model (sensitivity, not fixed 10-year endpoint) | Category D vs A+B | 2.44 (1.25-4.77); p=0.009 |
| Full recorded follow-up; age-group- and sex-adjusted Cox model (sensitivity, not fixed 10-year endpoint) | Per category increase (A=0 to D=3) | 1.63 (1.23-2.15); p<0.001 |

Privacy note: Exact individual age is not shared in the public dataset; age is represented only by prespecified age groups (<40, 40-60, >60 years).
