# Supplementary material for: Pragmatic cardiovascular-kidney-metabolic burden categories and 5-year all-cause mortality in Vietnamese outpatients: A retrospective cohort study
Source: PLoS One. 2026 Jul 22;21(7):e0354433. doi: 10.1371/journal.pone.0354433 (PMC13390809; doi:10.1371/journal.pone.0354433)
Supplement: Attachment — Submitted filename: Review comments.docx [file pone.0354433.s005.docx]

Dear Editor, Dear authors,

In this manuscript titled ***Pragmatic AHA-aligned cardiovascular-kidney-metabolic staging and 10-year allcause mortality in Vietnamese outpatients: a retrospective cohort study***, the overall workload is relatively light, including only the baseline characteristics of a cohort of 480 subjects and the analysis of the association between all-cause mortality and cardiovascular-kidney-metabolic (CKM) staging in 114 patients over a 10-year period. The main findings are presented in two tables and one figure. As a retrospective analysis, the overall level of evidence is low. In addition, the determination of whether a patient has “metabolic risk” and coronary artery disease (CAD) relies solely on outpatient records, which may be incomplete—thus substantially compromising the reliability of the conclusions. Therefore, major revision is required before this manuscript can be considered for publication in ***PLOS One***.

**Major comments:**

1. Line 73: Was the definition of "metabolic risk" self-defined by the authors' team, or was it based on criteria cited from established, relevant literature? Should this retrospective cohort have clear inclusion and exclusion criteria beyond the definitions of "metabolic risk", chronic kidney disease (CKD), and CAD?

2. According to the definition of the American Heart Association guidelines, the CKM syndrome staging system comprises stages 0, 1, 2, 3, and 4 (with stage 4 further subdivided into 4a and 4b), not stages 1-4 alone. In the guidelines, stage 0 refers to patients with no metabolic risk factors, CKD, or CAD, but the authors of this study defined such patients as "stage 1" (Line 78), raising concerns that the conclusions—should the manuscript be published—may be ambiguous and misleading.

3. Line 89: Is there a reasonable basis for combining the so-called "stage 1" and "stage 2" patients for analysis?

4. Given the relatively light overall workload, could the authors attempt stratification by age and add additional analyses to explore its value for specific age groups (e.g., < 40 years old, 40 to 60 years old, > 60 years old) in the "stage 2" and "stage 3" cohorts, where the overall number of deaths is comparatively larger?

5. The Conclusion section should include a few clear, emphatic sentences summarizing the main findings of the study. Best regards.

**Minor comments:**

1. With too many keywords, the core theme may become vague, and it is difficult for readers to quickly grasp the main point of the paper. And the standard words in the internationally recognized glossary such as MeSH should be preferred. Best regards.

2. In the note at the end of **Table 1**, the abbreviations in the table are not explained.

3. There are only 7 references, please add references to make the work more credible, and please update the references using literature from the last 5 years. Best regards.

4. It's great to see that the author team has an awareness of ethical review. May I ask if the team has conducted a standardized follow-up review of this research since 2015? If so, please provide the relevant documents.
