# Supplementary material for: Pragmatic cardiovascular-kidney-metabolic burden categories and 5-year all-cause mortality in Vietnamese outpatients: A retrospective cohort study
Source: PLoS One. 2026 Jul 22;21(7):e0354433. doi: 10.1371/journal.pone.0354433 (PMC13390809; doi:10.1371/journal.pone.0354433)
Supplement: Attachment — Submitted filename: CKM- reviewer comments.docx [file pone.0354433.s006.docx]

**Review Comments to the Author**

**General comments:** The manuscript addresses an important research question and contributes to a relevant gap in the current literature. Overall, the manuscript is well organized and logically structured. However, several issues should be addressed before the manuscript can be considered for publication. Firstly, the language requires substantial editing for grammar, syntax, and clarity. A thorough review by a native English speaker or a professional scientific editing service is recommended to improve readability and ensure precise communication of the study findings. Secondly, the statistical analysis could be strengthened. Please see the specific comments below for detailed concerns and recommendations.

**Materials and methods (line 54-61)** -There appears to be a discrepancy between the reported recruitment period (December 2015–May 2018), the date of dataset access (31 March 2024), and the reporting of 10-year mortality outcomes. As the available observation period does not appear sufficient to permit direct measurement of 10-year mortality for the entire cohort, the authors should clarify whether the reported 10-year mortality represents observed events, projected risk estimates, or model-based extrapolations. Detailed information regarding follow-up duration, censoring procedures, and the method used to derive the 10-year mortality estimates is required to assess the validity of the findings.

**Discussion (line 137-138)**-The assessment of subclinical coronary artery disease (CAD), a key component of CKM Stage 3, does not appear to have been included in the study. This may have resulted in the misclassification of some participants who were categorized as CKM Stage 2 but who may have met criteria for Stage 3 had subclinical CAD been systematically evaluated. This potential underestimation of Stage 3 prevalence may also have influenced the observed associations between CKM stages and mortality outcomes. In particular, the attenuation and loss of statistical significance of the association for Stage 3 after adjustment for age and sex may, at least in part, reflect the incomplete ascertainment of subclinical cardiovascular disease and consequent stage misclassification. I encourage the authors to acknowledge this limitation in the Discussion section and consider whether the absence of subclinical CAD data could have contributed to the lack of a consistent gradient of risk across CKM stages following multivariable adjustment.

**Line (158-160)**- same as conclusion, so can be omitted.

**References**- only 7 in number although many relation recent research articles are available online. Inclusion of valuable information from them may enrich the manuscript.

**Figure 1**: Figure 1 requires further refinement to improve its interpretability. The axes of the Kaplan–Meier survival curve are not adequately labelled, and the figure legend is incomplete. The authors should clearly specify the variables represented on both the x- and y-axes. In addition, the figure legend should report the log-rank test p-value used to compare survival across CKM categories. It would also be informative to include the numbers at risk at relevant time points for each CKM category beneath the Kaplan–Meier plot, as this is standard practice for the presentation of survival analyses and would facilitate interpretation of the findings.
