# Supplementary material for: Pragmatic cardiovascular-kidney-metabolic burden categories and 5-year all-cause mortality in Vietnamese outpatients: A retrospective cohort study
Source: PLoS One. 2026 Jul 22;21(7):e0354433. doi: 10.1371/journal.pone.0354433 (PMC13390809; doi:10.1371/journal.pone.0354433)
Supplement: Attachment — Submitted filename: 01_Response_to_Reviewers_FINAL.docx [file pone.0354433.s008.docx]

**Response to Editor and Reviewers**

Manuscript ID: PONE-D-26-11646

Revised title: Pragmatic cardiovascular-kidney-metabolic burden categories and 5-year all-cause mortality in Vietnamese outpatients: a retrospective cohort study

Dear Academic Editor and Reviewers,

We thank the Academic Editor and reviewers for their detailed and constructive comments. We have substantially revised the manuscript. The most important changes are summarized below:

We removed the fixed 10-year mortality claim and revised the primary endpoint to 5-year all-cause mortality, with administrative censoring at 5 years. This directly addresses the concern that recruitment from December 2015 to May 2018 and dataset access on 31 March 2024 do not support direct 10-year follow-up for every participant.

We replaced all “AHA-aligned CKM stage 1-4” terminology with “pragmatic CKM burden categories A-D.” We added a mapping table explaining that the categories are AHA-informed but are not official AHA CKM stages.

We reanalyzed the data using five-year logistic regression as the primary inferential model, complemented by Kaplan-Meier/log-rank and Cox time-to-event analyses. Figure 1 now has axis labels, a complete caption, log-rank p-value, and numbers at risk.

We clarified inclusion/exclusion criteria, the operational definition of documented metabolic-risk diagnosis, data access date, follow-up and censoring procedures, and handling of zero-event categories.

We added exploratory age-stratified summaries, updated the limitations regarding subclinical CAD and incomplete official CKM phenotyping, shortened/clarified the conclusion, added abbreviation notes to Table 1, and expanded the references with recent CKM literature.

We also updated the Data Availability Statement and the public Zenodo repository to the final version-specific DOI: 10.5281/zenodo.20499122. In response to PLOS human-participant data-sharing guidance, the public dataset has been further privacy-hardened: exact ages have been removed and replaced with prespecified age groups (<40, 40-60, >60 years), and the shared files contain no names, initials, contact information, hospital IDs, specific calendar dates, or other direct personal identifiers. The manuscript Table 1 now reports age only by age group.

Additional PLOS data policy comment. The journal noted that participant age could represent identifying human participant data and asked us to remove or anonymize the age information before public sharing.

Response: We agree and have revised both the manuscript and repository files. Exact individual age has been removed from the public dataset and replaced with prespecified age groups (<40, 40-60, and >60 years). Table 1 no longer reports a continuous age row; it now reports only age-group counts and percentages. The statistical analysis was updated so the reproducible public models adjust for age group and sex. We also removed specific calendar dates and direct identifiers from the public dataset. The Data Availability Statement now explicitly states that the Zenodo repository contains a privacy-hardened de-identified dataset and no names, initials, contact information, hospital identifiers, exact ages, or specific calendar dates.

# Responses to Reviewer #1

**Major comment** 1. Was the definition of “metabolic risk” self-defined, or based on literature? Should the retrospective cohort have clear inclusion/exclusion criteria beyond definitions of metabolic risk, CKD, and CAD?

Response: We agree. We now define the exposure as pragmatic CKM burden categories rather than official CKM stages. In the Methods, “documented metabolic-risk diagnosis” is explicitly defined as recorded hypertension, diabetes mellitus, and/or dyslipidemia at baseline. We explain that this operationalization is AHA-informed but limited to variables consistently available in the retrospective records. We also added clear analytic eligibility criteria: adult outpatients recruited during the approved period, baseline clinical/laboratory evaluation, complete variables required for CKM categorization, and recorded follow-up time/vital status.

**Major comment** 2. AHA CKM staging comprises stages 0, 1, 2, 3, and 4, not stages 1-4; defining patients with no risk factors as stage 1 may be misleading.

Response: We fully agree and have corrected this throughout. We no longer use numerical AHA CKM stage labels. The revised manuscript uses “pragmatic CKM burden categories A-D.” A new Methods table maps each category to its operational definition and explicitly states that these categories are not official AHA CKM stages and do not reproduce AHA stages 0-4 or 4a/4b.

**Major comment** 3. Is there a reasonable basis for combining the so-called stage 1 and stage 2 patients for analysis?

Response: We revised the terminology and now combine Categories A and B only for regression reference purposes. Category A included 24 participants and had zero five-year deaths. Keeping Category A as an independent referent would lead to zero-event instability and unreliable regression estimates. We therefore present Categories A and B separately in descriptive analyses and the Kaplan-Meier figure, but combine them as A+B for logistic and Cox regression.

**Major comment** 4. Could the authors attempt stratification by age and add additional analyses for specific age groups (<40, 40-60, >60 years)?

Response: We added exploratory age-stratified summaries in the Results and supporting analysis tables. There were no five-year deaths among participants aged <40 years. Among participants aged >60 years, mortality was highest in Category D (7/12, 58.3%). We did not emphasize formal stratum-specific models because several age-category cells had zero events, but the requested stratification is now reported.

**Major comment** 5. The Conclusion section should include a few clear, emphatic sentences summarizing the main findings.

Response: We rewrote the Conclusions to state the main findings directly: pragmatic CKM burden categories identified the highest absolute five-year mortality among participants with concomitant CKD and CAD, adjusted estimates were imprecise, and prospective validation with complete CKM phenotyping is needed.

**Minor comment** 1. Too many keywords; use standard internationally recognized glossary terms such as MeSH where possible.

Response: We reduced the keywords to six focused terms: cardiovascular-kidney-metabolic syndrome; chronic kidney disease; coronary artery disease; all-cause mortality; Vietnam; cohort study.

**Minor comment** 2. Abbreviations in Table 1 are not explained.

Response: We added a Table 1 note defining CAD, CKD, CKM, eGFR, hs-CRP, and LVEF.

**Minor comment** 3. There are only 7 references; add recent references from the last five years.

Response: We expanded and updated the references, including the AHA CKM advisory/scientific statement, JAMA/JAMA Network Open population studies, the Kailuan mortality study, the large Taiwanese PLoS Medicine study, and the KDIGO 2024 CKD guideline.

**Minor comment** 4. Was there a standardized follow-up review since 2015? If so, provide documents.

Response: We uploaded the original Vietnamese ethics approval and an English translation. The revised Methods clarify that the present work is a secondary analysis of de-identified data and involved no new participant contact or intervention. No separate institutional extension/follow-up approval document is available for the present secondary de-identified analysis. The original ethics approval covering the approved parent cohort period and an English translation have been uploaded, and the revised Methods clarify that no new participant contact or intervention was performed.

# Responses to Reviewer #2

General comment. The language requires substantial editing for grammar, syntax, and clarity; statistical analysis could be strengthened.

Response: We extensively edited the manuscript for clarity and reorganized the Methods, Results, and Discussion. We also restructured the statistical analysis around a valid fixed five-year endpoint, added administrative censoring details, used logistic regression as the primary inferential model, retained Kaplan-Meier/log-rank analyses for visualization, added Cox models as complementary analyses, and added sensitivity analyses. To align the public analysis with the privacy-hardened dataset, adjusted models now use age group and sex rather than exact individual age.

Materials and methods. There is a discrepancy between recruitment period, dataset access date, and reporting of 10-year mortality. Clarify whether 10-year mortality is observed, projected, or extrapolated; provide follow-up duration, censoring, and method.

Response: We agree. We removed the 10-year endpoint from the title, abstract, Methods, Results, Discussion, tables, and figure. The revised primary endpoint is five-year all-cause mortality, administratively censored at five years. This horizon is compatible with the recruitment end date and dataset access date. We explicitly state that we no longer present fixed 10-year mortality. The original full recorded follow-up analysis is retained only as a sensitivity analysis and is not interpreted as fixed 10-year mortality. The public dataset contains rounded follow-up times and no specific death or examination dates.

Discussion. Subclinical CAD, a key component of CKM Stage 3, was not assessed and could have caused misclassification and attenuation of associations.

Response: We agree and added this limitation. Because subclinical CAD, coronary calcium imaging, albuminuria, and noncoronary cardiovascular disease phenotyping were not available, some participants may have been under-classified. We specifically discuss that unmeasured subclinical CAD may have moved some participants from Category B to a higher official AHA CKM stage had full data been available, potentially attenuating differences between Categories B and C.

Lines 158-160 duplicate the conclusion and can be omitted.

Response: We removed the duplicate concluding sentence from the Discussion and retained a concise standalone Conclusions section.

References. Only 7 references are included; add recent research articles.

Response: We expanded the references with recent CKM studies and updated the CKD guideline citation to KDIGO 2024.

Figure 1. The Kaplan-Meier axes are inadequately labeled; legend is incomplete; include log-rank p-value and numbers at risk.

Response: We regenerated Figure 1. The revised figure includes x-axis label “Time since baseline (years),” y-axis label “Survival probability,” a category legend, log-rank p-value, and numbers at risk at 0, 1, 2, 3, 4, and 5 years.
