# Supplementary material for: Pragmatic cardiovascular-kidney-metabolic burden categories and 5-year all-cause mortality in Vietnamese outpatients: A retrospective cohort study
Source: PLoS One. 2026 Jul 22;21(7):e0354433. doi: 10.1371/journal.pone.0354433 (PMC13390809; doi:10.1371/journal.pone.0354433)
Supplement: Peer review history

# Peer review: Decision Letter 0

**DOI:** 10.1371/journal.pone.0354433.r001

**Authors:** Natural Hoi Sing Chu

1 Jun 2026

PONE-D-26-11646

Pragmatic AHA-aligned cardiovascular–kidney–metabolic staging and 10-year all-cause mortality in Vietnamese outpatients: a retrospective cohort study

PLOS One

Dear Dr. Nguyen,

Thank you for submitting your manuscript to PLOS ONE. After careful consideration, we feel that it has merit but does not fully meet PLOS ONE’s publication criteria as it currently stands. Therefore, we invite you to submit a revised version of the manuscript that addresses the points raised during the review process.

Please submit your revised manuscript by Jul 16 2026 11:59PM. If you will need more time than this to complete your revisions, please reply to this message or contact the journal office at plosone@plos.org. When you're ready to submit your revision, log on to https://www.editorialmanager.com/pone/ and select the 'Submissions Needing Revision' folder to locate your manuscript file.

Please include the following items when submitting your revised manuscript:

- A letter that responds to each point raised by the academic editor and reviewer(s). You should upload this letter as a separate file labeled 'Response to Reviewers'.
- A marked-up copy of your manuscript that highlights changes made to the original version. You should upload this as a separate file labeled 'Revised Manuscript with Track Changes'.
- An unmarked version of your revised paper without tracked changes. You should upload this as a separate file labeled 'Manuscript'.

If you would like to make changes to your financial disclosure, please include your updated statement in your cover letter. Guidelines for resubmitting your figure files are available below the reviewer comments at the end of this letter.

If applicable, we recommend that you deposit your laboratory protocols in protocols.io to enhance the reproducibility of your results. Protocols.io assigns your protocol its own identifier (DOI) so that it can be cited independently in the future. For instructions see: https://journals.plos.org/plosone/s/submission-guidelines#loc-laboratory-protocols. Additionally, PLOS ONE offers an option for publishing peer-reviewed Lab Protocol articles, which describe protocols hosted on protocols.io. Read more information on sharing protocols at https://plos.org/protocols?utm_medium=editorial-email&utm_source=authorletters&utm_campaign=protocols.

As the corresponding author, your ORCID iD is verified in the submission system and will appear in the published article. PLOS supports the use of ORCID, and we encourage all coauthors to register for an ORCID iD and use it as well. Please encourage your coauthors to verify their ORCID iD within the submission system before final acceptance, as unverified ORCID iDs will not appear in the published article. *Only*  the individual author can complete the verification step; PLOS staff *cannot*  verify ORCID iDs on behalf of authors.

We look forward to receiving your revised manuscript.

Kind regards,

Natural Hoi Sing Chu, Ph.D

Academic Editor

PLOS One

Journal Requirements:

When submitting your revision, we need you to address these additional requirements.

1. Please ensure that your manuscript meets PLOS ONE's style requirements, including those for file naming. The PLOS ONE style templates can be found at https://journals.plos.org/plosone/s/file?id=wjVg/PLOSOne_formatting_sample_main_body.pdf and https://journals.plos.org/plosone/s/file?id=ba62/PLOSOne_formatting_sample_title_authors_affiliations.pdf

If the reviewer comments include a recommendation to cite specific previously published works, please review and evaluate these publications to determine whether they are relevant and should be cited. There is no requirement to cite these works unless the editor has indicated otherwise.

[Note: HTML markup is below. Please do not edit.]

Reviewers' comments:

Reviewer's Responses to Questions

**Comments to the Author**

1. Is the manuscript technically sound, and do the data support the conclusions?

The manuscript must describe a technically sound piece of scientific research with data that supports the conclusions. Experiments must have been conducted rigorously, with appropriate controls, replication, and sample sizes. The conclusions must be drawn appropriately based on the data presented.

Reviewer #1: Partly

Reviewer #2: Partly

**********

2. Has the statistical analysis been performed appropriately and rigorously?

Reviewer #1: Yes

Reviewer #2: No

**********

3. Have the authors made all data underlying the findings in their manuscript fully available?

The PLOS Data policy requires authors to make all data underlying the findings described in their manuscript fully available without restriction, with rare exception (please refer to the Data Availability Statement in the manuscript PDF file). The data should be provided as part of the manuscript or its supporting information, or deposited to a public repository. For example, in addition to summary statistics, the data points behind means, medians and variance measures should be available. If there are restrictions on publicly sharing data—e.g. participant privacy or use of data from a third party—those must be specified.

Reviewer #1: Yes

Reviewer #2: Yes

**********

4. Is the manuscript presented in an intelligible fashion and written in standard English?

PLOS ONE does not copyedit accepted manuscripts, so the language in submitted articles must be clear, correct, and unambiguous. Any typographical or grammatical errors should be corrected at revision, so please note any specific errors here.

Reviewer #1: Yes

Reviewer #2: Yes

**********

5. Review Comments to the Author

Please use the space provided to explain your answers to the questions above. You may also include additional comments for the author, including concerns about dual publication, research ethics, or publication ethics. (Please upload your review as an attachment if it exceeds 20,000 characters)

Reviewer #1: Major comments:

1. Line 73: Was the definition of "metabolic risk" self-defined by the authors' team, or was it based on criteria cited from established, relevant literature? Should this retrospective cohort have clear inclusion and exclusion criteria beyond the definitions of "metabolic risk", chronic kidney disease (CKD), and CAD?

2. According to the definition of the American Heart Association guidelines, the CKM syndrome staging system comprises stages 0, 1, 2, 3, and 4 (with stage 4 further subdivided into 4a and 4b), not stages 1-4 alone. In the guidelines, stage 0 refers to patients with no metabolic risk factors, CKD, or CAD, but the authors of this study defined such patients as "stage 1" (Line 78), raising concerns that the conclusions—should the manuscript be published—may be ambiguous and misleading.

3. Line 89: Is there a reasonable basis for combining the so-called "stage 1" and "stage 2" patients for analysis?

4. Given the relatively light overall workload, could the authors attempt stratification by age and add additional analyses to explore its value for specific age groups (e.g., < 40 years old, 40 to 60 years old, > 60 years old) in the "stage 2" and "stage 3" cohorts, where the overall number of deaths is comparatively larger?

5. The Conclusion section should include a few clear, emphatic sentences summarizing the main findings of the study. Best regards.

Minor comments:

1. With too many keywords, the core theme may become vague, and it is difficult for readers to quickly grasp the main point of the paper. And the standard words in the internationally recognized glossary such as MeSH should be preferred. Best regards.

2. In the note at the end of Table 1, the abbreviations in the table are not explained.

3. There are only 7 references, please add references to make the work more credible, and please update the references using literature from the last 5 years. Best regards.

4. It's great to see that the author team has an awareness of ethical review. May I ask if the team has conducted a standardized follow-up review of this research since 2015? If so, please provide the relevant documents.

Reviewer #2: General comments: The manuscript addresses an important research question and contributes to a relevant gap in the current literature. Overall, the manuscript is well organized and logically structured. However, several issues should be addressed before the manuscript can be considered for publication. Firstly, the language requires substantial editing for grammar, syntax, and clarity. A thorough review by a native English speaker or a professional scientific editing service is recommended to improve readability and ensure precise communication of the study findings. Secondly, the statistical analysis could be strengthened. Please see the specific comments below for detailed concerns and recommendations.

Please also find the attachment.

**********

6. PLOS authors have the option to publish the peer review history of their article (what does this mean?). If published, this will include your full peer review and any attached files.

If you choose “no”, your identity will remain anonymous but your review may still be made public.

**Do you want your identity to be public for this peer review?**  For information about this choice, including consent withdrawal, please see our Privacy Policy.

Reviewer #1: No

Reviewer #2: **Yes:** PONKAJ KANTI DATTA

**********

[NOTE: If reviewer comments were submitted as an attachment file, they will be attached to this email and accessible via the submission site. Please log into your account, locate the manuscript record, and check for the action link "View Attachments". If this link does not appear, there are no attachment files.]

To ensure your figures meet our technical requirements, please review our figure guidelines: https://journals.plos.org/plosone/s/figures

You may also use PLOS’s free figure tool, NAAS, to help you prepare publication quality figures: https://journals.plos.org/plosone/s/figures#loc-tools-for-figure-preparation.

NAAS will assess whether your figures meet our technical requirements by comparing each figure against our figure specifications.

Attachment Submitted filename: Review comments.docx

Attachment Submitted filename: CKM- reviewer comments.docx

# Author comment: Author response to Decision Letter 1

**DOI:** 10.1371/journal.pone.0354433.r002

2 Jun 2026

Dear Academic Editor and Reviewers,

We thank the Academic Editor and reviewers for their detailed and constructive comments. We have substantially revised the manuscript.

The most important changes are summarized below:

1. We removed the fixed 10-year mortality claim and revised the primary endpoint to 5-year all-cause mortality, with administrative censoring at 5 years. This directly addresses the concern that recruitment from December 2015 to May 2018 and dataset access on 31 March 2024 do not support direct 10-year follow-up for every participant.

2. We replaced all “AHA-aligned CKM stage 1-4” terminology with “pragmatic CKM burden categories A-D.” We added a mapping table explaining that the categories are AHA-informed but are not official AHA CKM stages.

3. We reanalyzed the data using five-year logistic regression as the primary inferential model, complemented by Kaplan-Meier/log-rank and Cox time-to-event analyses. Figure 1 now has axis labels, a complete caption, log-rank p-value, and numbers at risk.

4. We clarified inclusion/exclusion criteria, the operational definition of documented metabolic-risk diagnosis, data access date, follow-up and censoring procedures, and handling of zero-event categories.

5. We added exploratory age-stratified summaries, updated the limitations regarding subclinical CAD and incomplete official CKM phenotyping, shortened and clarified the conclusion, added abbreviation notes to Table 1, and expanded the references with recent CKM literature.

6. We updated the Data Availability Statement and the public Zenodo repository to the final version-specific DOI: 10.5281/zenodo.20499122. In response to PLOS human-participant data-sharing guidance, the public dataset has been further privacy-hardened: exact ages have been removed and replaced with prespecified age groups (<40, 40-60, >60 years), and the shared files contain no names, initials, contact information, hospital IDs, specific calendar dates, or other direct personal identifiers. The manuscript Table 1 now reports age only by age group.

Additional PLOS data policy comment:

The journal noted that participant age could represent identifying human participant data and asked us to remove or anonymize age information before public sharing.

Response:

We agree and have revised both the manuscript and repository files. Exact individual age has been removed from the public dataset and replaced with prespecified age groups (<40, 40-60, and >60 years). Table 1 no longer reports a continuous age row; it now reports only age-group counts and percentages. The statistical analysis was updated so the reproducible public models adjust for age group and sex. We also removed specific calendar dates and direct identifiers from the public dataset. The Data Availability Statement now explicitly states that the Zenodo repository contains a privacy-hardened de-identified dataset and no names, initials, contact information, hospital identifiers, exact ages, or specific calendar dates.

Responses to Reviewer #1

Major comment 1. Was the definition of “metabolic risk” self-defined, or based on literature? Should the retrospective cohort have clear inclusion/exclusion criteria beyond definitions of metabolic risk, CKD, and CAD?

Response:

We agree. We now define the exposure as pragmatic CKM burden categories rather than official CKM stages. In the Methods, “documented metabolic-risk diagnosis” is explicitly defined as recorded hypertension, diabetes mellitus, and/or dyslipidemia at baseline. We explain that this operationalization is AHA-informed but limited to variables consistently available in the retrospective records. We also added clear analytic eligibility criteria: adult outpatients recruited during the approved period, baseline clinical/laboratory evaluation, complete variables required for CKM categorization, and recorded follow-up time/vital status.

Major comment 2. AHA CKM staging comprises stages 0, 1, 2, 3, and 4, not stages 1-4; defining patients with no risk factors as stage 1 may be misleading.

Response:

We fully agree and have corrected this throughout. We no longer use numerical AHA CKM stage labels. The revised manuscript uses “pragmatic CKM burden categories A-D.” A new Methods table maps each category to its operational definition and explicitly states that these categories are not official AHA CKM stages and do not reproduce AHA stages 0-4 or 4a/4b.

Major comment 3. Is there a reasonable basis for combining the so-called stage 1 and stage 2 patients for analysis?

Response:

We revised the terminology and now combine Categories A and B only for regression reference purposes. Category A included 24 participants and had zero five-year deaths. Keeping Category A as an independent referent would lead to zero-event instability and unreliable regression estimates. We therefore present Categories A and B separately in descriptive analyses and the Kaplan-Meier figure, but combine them as A+B for logistic and Cox regression.

Major comment 4. Could the authors attempt stratification by age and add additional analyses for specific age groups (<40, 40-60, >60 years)?

Response:

We added exploratory age-stratified summaries in the Results and supporting analysis tables. There were no five-year deaths among participants aged <40 years. Among participants aged >60 years, mortality was highest in Category D (7/12, 58.3%). We did not emphasize formal stratum-specific models because several age-category cells had zero events, but the requested stratification is now reported.

Major comment 5. The Conclusion section should include a few clear, emphatic sentences summarizing the main findings.

Response:

We rewrote the Conclusions to state the main findings directly: pragmatic CKM burden categories identified the highest absolute five-year mortality among participants with concomitant CKD and CAD, adjusted estimates were imprecise, and prospective validation with complete CKM phenotyping is needed.

Minor comment 1. Too many keywords; use standard internationally recognized glossary terms such as MeSH where possible.

Response:

We reduced the keywords to six focused terms: cardiovascular-kidney-metabolic syndrome; chronic kidney disease; coronary artery disease; all-cause mortality; Vietnam; cohort study.

Minor comment 2. Abbreviations in Table 1 are not explained.

Response:

We added a Table 1 note defining CAD, CKD, CKM, eGFR, hs-CRP, and LVEF.

Minor comment 3. There are only 7 references; add recent references from the last five years.

Response:

We expanded and updated the references, including the AHA CKM advisory/scientific statement, JAMA/JAMA Network Open population studies, the Kailuan mortality study, the large Taiwanese PLoS Medicine study, and the KDIGO 2024 CKD guideline.

Minor comment 4. Was there a standardized follow-up review since 2015? If so, provide documents.

Response:

We uploaded the original Vietnamese ethics approval and an English translation. The revised Methods clarify that the present work is a secondary analysis of de-identified data and involved no new participant contact or intervention. No separate institutional extension/follow-up approval document is available for the present secondary de-identified analysis. The original ethics approval covering the approved parent cohort period and an English translation have been uploaded.

Responses to Reviewer #2

General comment. The language requires substantial editing for grammar, syntax, and clarity; statistical analysis could be strengthened.

Response:

We extensively edited the manuscript for clarity and reorganized the Methods, Results, and Discussion. We also restructured the statistical analysis around a valid fixed five-year endpoint, added administrative censoring details, used logistic regression as the primary inferential model, retained Kaplan-Meier/log-rank analyses for visualization, added Cox models as complementary analyses, and added sensitivity analyses. To align the public analysis with the privacy-hardened dataset, adjusted models now use age group and sex rather than exact individual age.

Materials and methods. There is a discrepancy between recruitment period, dataset access date, and reporting of 10-year mortality. Clarify whether 10-year mortality is observed, projected, or extrapolated; provide follow-up duration, censoring, and method.

Response:

We agree. We removed the 10-year endpoint from the title, abstract, Methods, Results, Discussion, tables, and figure. The revised primary endpoint is five-year all-cause mortality, administratively censored at five years. This horizon is compatible with the recruitment end date and dataset access date. We explicitly state that we no longer present fixed 10-year mortality. The original full recorded follow-up analysis is retained only as a sensitivity analysis and is not interpreted as fixed 10-year mortality. The public dataset contains rounded follow-up times and no specific death or examination dates.

Discussion. Subclinical CAD, a key component of CKM Stage 3, was not assessed and could have caused misclassification and attenuation of associations.

Response:

We agree and added this limitation. Because subclinical CAD, coronary calcium imaging, albuminuria, and noncoronary cardiovascular disease phenotyping were not available, some participants may have been under-classified. We specifically discuss that unmeasured subclinical CAD may have moved some participants from Category B to a higher official AHA CKM stage had full data been available, potentially attenuating differences between Categories B and C.

Lines 158-160 duplicate the conclusion and can be omitted.

Response:

We removed the duplicate concluding sentence from the Discussion and retained a concise standalone Conclusions section.

References. Only 7 references are included; add recent research articles.

Response:

We expanded the references with recent CKM studies and updated the CKD guideline citation to KDIGO 2024.

Figure 1. The Kaplan-Meier axes are inadequately labeled; legend is incomplete; include log-rank p-value and numbers at risk.

Response:

We regenerated Figure 1. The revised figure includes x-axis label “Time since baseline (years),” y-axis label “Survival probability,” a category legend, log-rank p-value, and numbers at risk at 0, 1, 2, 3, 4, and 5 years.

Attachment Submitted filename: 01_Response_to_Reviewers_FINAL.docx

# Peer review: Decision Letter 1

**DOI:** 10.1371/journal.pone.0354433.r003

**Authors:** Natural Hoi Sing Chu

7 Jul 2026

Pragmatic cardiovascular-kidney-metabolic burden categories and 5-year all-cause mortality in Vietnamese outpatients: a retrospective cohort study

PONE-D-26-11646R1

Dear Dr. Nguyen,

We’re pleased to inform you that your manuscript has been judged scientifically suitable for publication and will be formally accepted for publication once it meets all outstanding technical requirements.

Within one week, you’ll receive an e-mail detailing the required amendments. When these have been addressed, you’ll receive a formal acceptance letter and your manuscript will be scheduled for publication.

An invoice will be generated when your article is formally accepted. Please note, if your institution has a publishing partnership with PLOS and your article meets the relevant criteria, all or part of your publication costs will be covered. Please make sure your user information is up-to-date by logging into Editorial Manager at Editorial Manager® and clicking the ‘Update My Information' link at the top of the page. For questions related to billing, please contact billing support.

If your institution or institutions have a press office, please notify them about your upcoming paper to help maximize its impact. If they’ll be preparing press materials, please inform our press team as soon as possible -- no later than 48 hours after receiving the formal acceptance. Your manuscript will remain under strict press embargo until 2 pm Eastern Time on the date of publication. For more information, please contact onepress@plos.org.

Kind regards,

Natural Hoi Sing Chu, Ph.D

Academic Editor

PLOS One

Additional Editor Comments (optional):

Reviewers' comments:

Reviewer's Responses to Questions

**Comments to the Author**

1. If the authors have adequately addressed your comments raised in a previous round of review and you feel that this manuscript is now acceptable for publication, you may indicate that here to bypass the “Comments to the Author” section, enter your conflict of interest statement in the “Confidential to Editor” section, and submit your "Accept" recommendation.

Reviewer #1: All comments have been addressed

Reviewer #2: All comments have been addressed

**********

2. Is the manuscript technically sound, and do the data support the conclusions?

The manuscript must describe a technically sound piece of scientific research with data that supports the conclusions. Experiments must have been conducted rigorously, with appropriate controls, replication, and sample sizes. The conclusions must be drawn appropriately based on the data presented.

Reviewer #1: Yes

Reviewer #2: Yes

**********

3. Has the statistical analysis been performed appropriately and rigorously?

Reviewer #1: Yes

Reviewer #2: Yes

**********

4. Have the authors made all data underlying the findings in their manuscript fully available?

The PLOS Data policy requires authors to make all data underlying the findings described in their manuscript fully available without restriction, with rare exception (please refer to the Data Availability Statement in the manuscript PDF file). The data should be provided as part of the manuscript or its supporting information, or deposited to a public repository. For example, in addition to summary statistics, the data points behind means, medians and variance measures should be available. If there are restrictions on publicly sharing data—e.g. participant privacy or use of data from a third party—those must be specified.

Reviewer #1: Yes

Reviewer #2: Yes

**********

5. Is the manuscript presented in an intelligible fashion and written in standard English?

PLOS ONE does not copyedit accepted manuscripts, so the language in submitted articles must be clear, correct, and unambiguous. Any typographical or grammatical errors should be corrected at revision, so please note any specific errors here.

Reviewer #1: Yes

Reviewer #2: Yes

**********

6. Review Comments to the Author

Please use the space provided to explain your answers to the questions above. You may also include additional comments for the author, including concerns about dual publication, research ethics, or publication ethics. (Please upload your review as an attachment if it exceeds 20,000 characters)

Reviewer #1: (No Response)

Reviewer #2: Thank you for the revision work.

Minor change request: In the text- outcome and follow up , line 94 and Discussion, line 163 , the sentences are directed towards the reviewer. Please write to the readers. No need to mention that you have replaced 10 year mortality with 5 year mortality. Please present to the reader that you are reporting 5 year mortality. Thank you.

**********

7. PLOS authors have the option to publish the peer review history of their article (what does this mean?). If published, this will include your full peer review and any attached files.

If you choose “no”, your identity will remain anonymous but your review may still be made public.

**Do you want your identity to be public for this peer review?**  For information about this choice, including consent withdrawal, please see our Privacy Policy.

Reviewer #1: **Yes:** Yuqing Zhu

Reviewer #2: No

**********

# Editor report: Acceptance letter

**DOI:** 10.1371/journal.pone.0354433.r004

**Authors:** Natural Hoi Sing Chu

PONE-D-26-11646R1

PLOS One

Dear Dr. Nguyen,

I'm pleased to inform you that your manuscript has been deemed suitable for publication in PLOS One. Congratulations! Your manuscript is now being handed over to our production team.

At this stage, our production department will prepare your paper for publication. This includes ensuring the following:

* All references, tables, and figures are properly cited

* All relevant supporting information is included in the manuscript submission,

* There are no issues that prevent the paper from being properly typeset

You will receive further instructions from the production team, including instructions on how to review your proof when it is ready. Please keep in mind that we are working through a large volume of accepted articles, so please give us a few days to review your paper and let you know the next and final steps.

Lastly, if your institution or institutions have a press office, please let them know about your upcoming paper now to help maximize its impact. If they'll be preparing press materials, please inform our press team within the next 48 hours. Your manuscript will remain under strict press embargo until 2 pm Eastern Time on the date of publication. For more information, please contact onepress@plos.org.

You will receive an invoice from PLOS for your publication fee after your manuscript has reached the completed accept phase. If you receive an email requesting payment before acceptance or for any other service, this may be a phishing scheme. Learn how to identify phishing emails and protect your accounts at https://explore.plos.org/phishing.

If we can help with anything else, please email us at customercare@plos.org.

Thank you for submitting your work to PLOS ONE and supporting open access.

Kind regards,

PLOS ONE Editorial Office Staff

on behalf of

Dr. Natural Hoi Sing Chu

Academic Editor

PLOS One
